# The Impact of the Epithelial–Mesenchymal Transition Regulator Hepatocyte Growth Factor Receptor/Met on Skin Immunity by Modulating Langerhans Cell Migration

**DOI:** 10.3389/fimmu.2018.00517

**Published:** 2018-03-16

**Authors:** Zsofia Sagi, Thomas Hieronymus

**Affiliations:** ^1^Department of Cell Biology, Institute of Biomedical Engineering, RWTH Aachen University Medical School, Aachen, Germany; ^2^Helmholtz Institute for Biomedical Engineering, RWTH Aachen University, Aachen, Germany

**Keywords:** Langerhans cell, dendritic cell, Met signaling, hepatocyte growth factor, epithelial–mesenchymal transition, skin injury, immunity, tolerance

## Abstract

Langerhans cells (LCs), the epidermal dendritic cell (DC) subset, express the transmembrane tyrosine kinase receptor Met also known as hepatocyte growth factor (HGF) receptor. HGF is the exclusive ligand of Met and upon binding executes mitogenic, morphogenic, and motogenic activities to various cells. HGF exerts anti-inflammatory activities *via* Met signaling and was found to regulate various functions of immune cells, including differentiation and maturation, cytokine production, cellular migration and adhesion, and T cell effector function. It has only recently become evident that a number of HGF-regulated functions in inflammatory processes and immune responses are imparted *via* DCs. However, the mechanisms by which Met signaling in DCs conveys its immunoregulatory effects have not yet been fully understood. In this review, we focus on the current knowledge of Met signaling in DCs with particular attention on the morphogenic and motogenic activities. Met signaling was shown to promote DC mobility by regulating matrix metalloproteinase activities and adhesion. This is a striking resemblance to the role of Met in regulating a cell fate program during embryonic development, wound healing, and in tumor invasion known as epithelial–mesenchymal transition (EMT). Hence, we propose the concept that an EMT program is executed by Met signaling in LCs.

## Introduction

Name giving, hepatocyte growth factor (HGF) was initially identified as a mitogenic factor for rat hepatocytes (1, 2). However, it then became evident that HGF elicits various biological activities in a number of different cell types. Independent studies before cloning of the HGF gene identified the same molecule as a potent inducer of epithelial cell motility (and thus termed as scatter factor) (3) and as a fibroblast-derived cytotoxic factor for some tumor cell lines (4). Furthermore, HGF was found to promote cell survival, tissue protection and regeneration but restrain fibrosis and inflammation (5). All these activities are commenced by binding of HGF to its cognate receptor Met, which was originally identified as a transforming oncogene (6, 7). HGF is primarily secreted by mesenchymal cells that are frequently positioned in the immediate vicinity of Met-expressing cells reflecting the limited capacity of HGF to diffuse *in vivo* (8). The indispensable roles of Met signaling by HGF for embryonic development and tissue regeneration have been demonstrated by targeted disruption of the HGF and Met genes. Both the conventional Met- or HGF-null mutations in mice result in a lethal phenotype *in utero* caused by the impaired development of placenta and liver (9–11). In addition, Met-expressing myogenic precursors fail to emigrate from the dermomyotome leading to the total absence of all muscle groups derived from these migratory progenitor cells (10, 12). The contribution of Met signaling to the long-range migration of cells during development is mediated by induction of a cell fate program known as epithelial–mesenchymal transition (EMT) that is fundamental not only during embryogenesis but also in tissue regeneration of the adult organism (8, 13, 14).

A corresponding aberrant activation of the EMT program by Met signaling during tumorgenesis results in invasive growth and metastasis of tumor cells (8, 13, 14). The oncogenic role and potential interventions of the Met signaling pathway for tumor therapy have long been a major focus of research, which is comprehensively documented in a number of excellent reviews (5, 15–18). However, a growing body of evidence suggests an additionally important role of Met signaling in control of immune cell functions and thus in regulation of immunity. Here, we will discuss the current understanding of Met signaling and function in dendritic cells (DCs) with particular emphasis on the motogenic activities of Met for Langerhans cells (LCs).

## Structural and Functional Features of HGF and MET

### Hepatocyte Growth Factor

**Figure 1:**
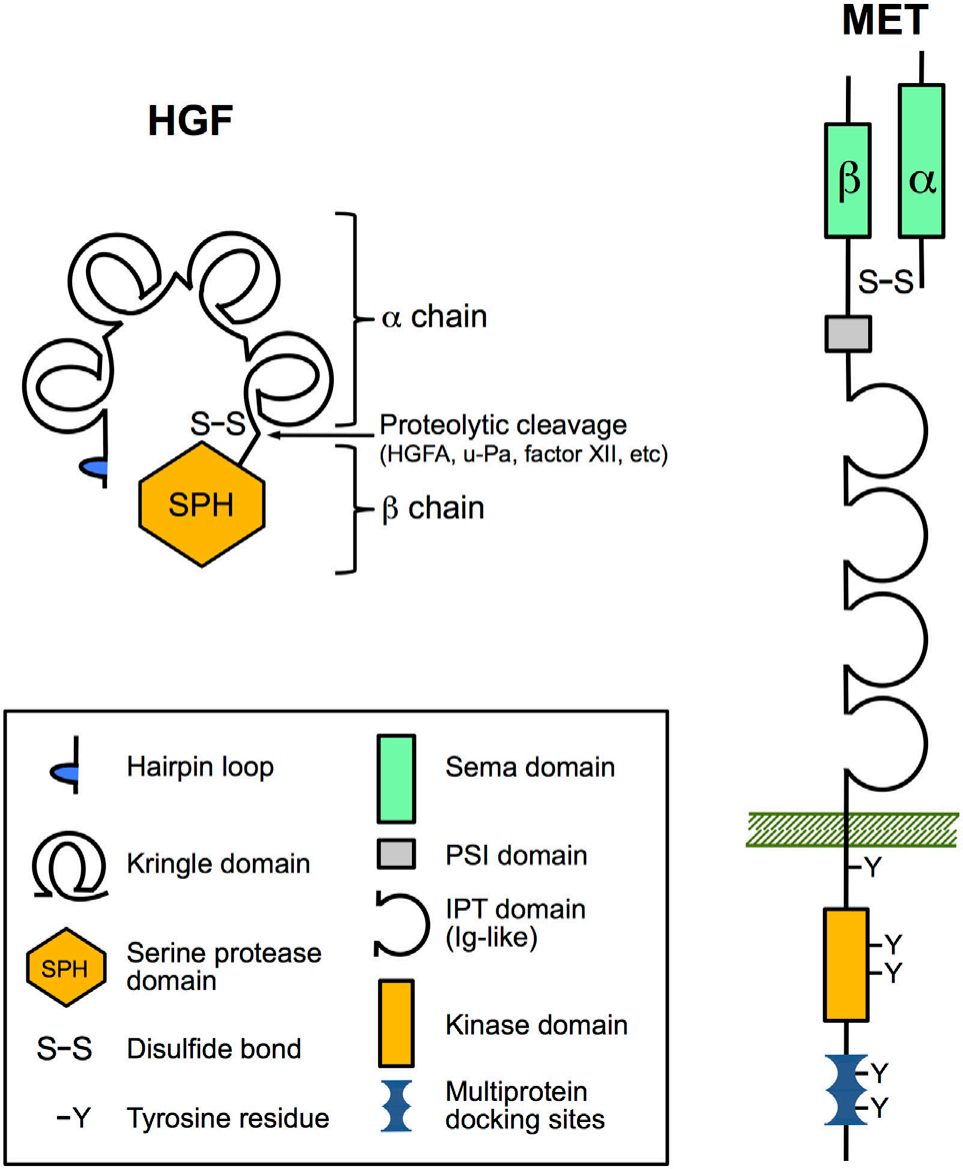
The domain structure of hepatocyte growth factor (HGF) and Met. HGF and Met are both synthesized as inert single-chain precursors and are cleaved to generate mature disulfide-linked α–β heterodimers that have signaling competence. The α-chain of HGF contains an N-terminal hairpin loop and four kringle domains, and the β-chain harbors a catalytically inactive serine proteinase homology domain. Important for ligand binding the α-chain of Met and the amino-terminal end of the β-chain form a so-called Sema domain found in semaphorin axon-guidance proteins and plexins (cell adhesion and semaphorin receptors). The remainder of the extracellular part of the β-chain contains a PSI domain (present in plexins, semaphorins, and integrins) and four IPT domains (immunoglobulin-like fold shared by plexins and transcription factors). IPT3 and IPT4 serve as the high-affinity docking site for HGF. The cytoplasmic region comprises the tyrosine kinase domain, a juxtamembrane regulatory region, and a multiprotein-docking site at the carboxy terminus essential for downstream signaling.

Biologically active HGF is a disulfide-linked heterodimeric molecule composed of a 69 kDa α-chain and a 34 kDa β-chain that is derived from an inactive single-chain precursor (pro-HGF; Figure 1) (5). HGF and the structurally similar cytokine macrophage-stimulating protein (MST1, also known as HGF-like or MSP) comprise the unique group of plasminogen-like cytokines (19). Unlike other cytokines and growth factors, they share structural homologies with coagulation factors, including prothrombin, coagulation factor XII, plasminogen and plasminogen activators (urokinase type, u-Pa and tissue type, t-Pa), and HGF activator protein (HGFA). They have in common the presence of a serine proteinase homology (SPH) domain and at least one kringle domain. HGF and MST1 have lost the proteinase activity due to loss of catalytic residues in the SPH domain but retained the requirement for proteolytic cleavage to become mature proteins (20, 21). The α-chain of HGF with four kringle domains confers high-affinity binding to the Met receptor and its dimerization, while subsequent binding of the β-chain is required for the activation of Met signaling (22).

A complex process is regulating the availability of bioactive HGF. Both pro-HGF and cleaved HGF bind with high affinity to heparan sulfate proteoglycans that limits the diffusion and leads to accumulation within the extracellular matrix (ECM) (23). Pro-HGF can be cleaved by many serine proteinases present in serum or cell membrane including u-Pa, t-Pa, plasma kallikrein, factor XII, HGFA, and others (5). Among them, HGFA is one of the most efficient in processing pro-HGF. Again, HGFA like other serine proteinases of this family is synthesized as an inactive single-chain precursor (pro-HGFA) that needs proteolytic cleavage, e.g., by the central coagulation factor thrombin to become an active proteinase (24). The contribution of the coagulation cascade to the activation of HGF strongly points toward the significant role of HGF in tissue injury (25). In addition, the activity of HGFA is regulated by specific inhibitors of the Kunitz-type family of membrane-bound serine protease inhibitors, namely, HGFA inhibitor 1 (HAI-1) and HAI-2 (26). HGFA activity is suppressed by binding to HAI-1 on the cell surface. The HGFA/HAI-1 complexes on the cell surface can be released by metalloproteinase-mediated shedding of the HAI-1 ectodomain (5). Interestingly, DCs also express HAI-1 and thus may themselves be capable of regulating the availability of bioactive HGF in injured tissue and inflammation (27). Notably, pro-inflammatory mediators, including IL-1β and prostaglandin E2, are potent inducers of HGF expression and cleavage of HAI-1 (5). The regulatory mechanisms that control HGF activity thus refer to the mutual link of inflammation with tissue damage and regeneration.

### Met

All biological functions of HGF are exerted by binding to its unique receptor Met. Like its ligand, Met is synthesized as an immature single-chain precursor that is cleaved by intracellular endoproteinases to form the mature membrane-bound disulfide-linked α-β heterodimer (Figure 1). Met shares similar structural features with the receptor for MST1 (MST1R; also known as Ron/CD136 or as STK in mice) (28, 29). The mature form of Met comprises the extracellular 50 kDa α-chain and the transmembrane-passing 145 kDa β-chain. The so-called Sema domain constituted by the α-chain and the amino-terminal end of the β-chain is required for HGF binding and activation of Met signaling (30). Upon HGF binding, Met undergoes dimerization and autophosphorylation of two critical tyrosine residues in the activation loop of the kinase domain, leading to enhanced catalytic activity (14). Further phosphorylation of tyrosine residues within the C-terminal docking site controls recruitment of various signaling and adaptor proteins that in turn can activate downstream signaling pathways, such as ERK, AKT, and RAC1 pathways (8, 14, 31).

Met interacts with other cell surface receptor that can modulate Met signaling in ligand-dependent and -independent manner. Remarkably, this includes a number of surface receptors involved in regulation of cellular motility and migration, such as integrin α6β4 (32), plexin-B1 (33), CD44 (34, 35), Mif receptor (36), and E-cadherin (37). Met and plexins share the highly homologous Sema domain that allows physical interactions between them (38). Consequently, semaphorin binding to plexins can lead to Met transactivation independent from HGF binding (14). The expression of different members of the plexin family on DCs and their contribution to the regulation of DC migration has already been described (39–41), suggesting that this may involve interaction with Met signaling.

## The Immunoregulatory Function of HGF/MET Signaling

Beyond the well-recognized role of Met signaling in epithelial cells and tumor development, early reports already provided evidence for a role in hematopoietic cells (42, 43), and accumulating data from recent years clearly demonstrated important functions in hematopoiesis and immunity.

Constitutive Met expression is limited to hematopoietic progenitor cells and their antigen-presenting progenies, including B cells, monocytes/macrophages, and DCs (29, 44). However, exposure to pro-inflammatory cytokines leads to induction and/or upregulation of Met expression in various cell types, again pointing toward the regulatory link of tissue injury to the inflammatory response. Indeed, recent findings suggest conditionally inducible expression of Met in other immune cells including neutrophils (45) and a specific subset of CD8+ T cells (46). Interestingly, in neutrophils, the Met expression was found to be required for chemoattraction in response to HGF and transmigration across an endothelial barrier (45). This provides further evidence that Met signaling can exert motogenic functions in immune cells. Met signaling was reported to play a role in regulating B cell homing to lymph nodes (LNs) (47) and was identified as a potent inducer of directional migration in monocytes (48, 49). Likewise, Met expression was found to regulate splenic DC function (50, 51) and was further shown to be expressed on bone marrow (BM)-derived DCs, dermal DCs, and LCs (52, 53).

**Figure 2:**
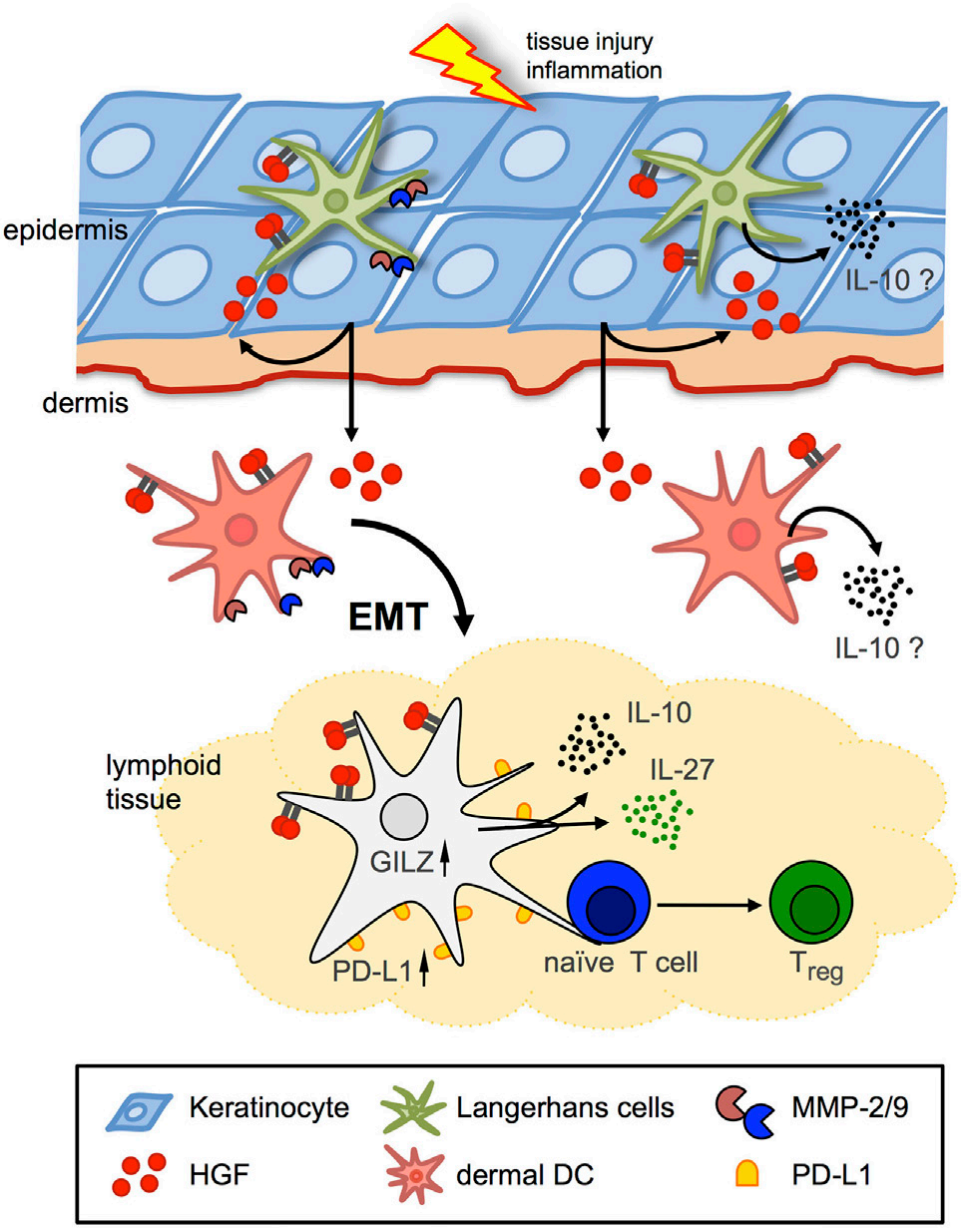
Hepatocyte growth factor (HGF)/Met signaling in Langerhans cells (LCs)/dendritic cells (DCs). Schematic representation of two major avenues of Met signaling on DCs in peripheral and lymphoid tissues. Met signaling induces LC and dermal DC emigration from skin in an epithelial–mesenchymal transition (EMT)-like process, including matrix metalloproteinase (MMP) activation to facilitate arrival to draining lymph nodes and antigen presentation to naive T cells. HGF induces tolerogenic phenotypes by IL-10 and IL-27 secretion and upregulated expression of, e.g., glucocorticoid-induced leucine zipper (GILZ) and programmed-death ligand 1 (PD-L1) in DCs, which eventually results in enhanced numbers of regulatory T cells (T_regs_).

For the Met ligand HGF, it has been shown that it—frequently in synergy with other growth factors—can support erythropoiesis, thrombopoiesis, and myelopoiesis and development of Met-expressing thymocytes (5, 43). Fibroblast-like stromal cells in lymphoid tissues including spleen (54), LNs (47), thymus (55), and BM (56) constitutively produce HGF that can be modulated by activated T cells (54). HGF might thus play additional roles within lymphoid organs on Met-expressing cells, such as regulation of cell survival (36) and cytokine production (50, 51, 57–59), thereby influencing immune responses (Figure 2). Moreover, also hematopoietic cells including platelets, mast cells, neutrophils, and macrophages can produce HGF.

In response to infection or tissue injury the production of HGF is further stimulated by pro-inflammatory cytokines, including IL-1α, IL-1β, TNF-α, and IL-6 (60, 61). By contrast, anti-inflammatory factors, such as glucocorticoids (62), 1,25-dihydroxyvitamin D3 (63), and TGF-β (64), inhibit HGF production. This points toward a potential pro-inflammatory role of HGF. In line with this concept, stimulation of monocytes with HGF induced upregulation of pro-inflammatory factors, including IL-4, IL-1β, GM-CSF, and MIP-1β (48). This notion was further fueled by studies, which showed that HGF stimulated the antigen presentation capacity of adult human blood monocytes (65) and that in the murine experimental autoimmune encephalomyelitis (EAE) model Met signaling promoted the development of M1 macrophages fostering inflammation (66).

In addition to its pro-inflammatory role, it has been proposed that HGF exerts anti-inflammatory activities (5). Indeed, HGF was found to grant protective effects in various animal models of inflammatory diseases, including collagen-induced arthritis (58), chronic airway inflammation (50), inflammatory bowel disease (67, 68), and even in EAE in contrast to the previously cited study (51, 69) by regulating various functions of immune cells, including cytokine production, migration, and adhesion. A number of these studies provided strong evidence that HGF-mediated immunomodulatory effects were imparted *via* direct impact on DC function (Figure 2). HGF was shown to impair DC activation resulting in an obstructed antigen-presenting capacity (50, 70), and that HGF inhibited immunogenic DC function by stimulating IL-10 secretion (59), which leads to suppression of the DC function in an autocrine manner (71). In the EAE model, HGF was shown to confer DCs with suppressive competence resulting in induction of regulatory T cells (T_regs_) (51, 59, 69). Noteworthy, HGF treatment of DCs was found to increase expression of programmed-death ligand 1 and IL-27, which are potent factors to mediate DC-driven generation of T_regs_ (69). In addition, DCs exhibited increased glucocorticoid-induced leucine zipper (GILZ) expression upon HGF stimulation. Notably, previous studies revealed GILZ expression to be a shared feature of tolerogenic DCs induced by IL-10, TGF-β, and glucocorticoids (72, 73) (which in turn can regulate HGF expression; see above). These results strongly indicate that HGF exerts immunoregulatory activities directly through Met-dependent regulation of DC function. However, an immunoregularory function of HGF/Met signaling in skin immunity has been scarcely explored.

## MET Signaling in Skin Injury and the Impact on LC Migration

The skin represents one of the largest organs of the human body that also establishes a direct interface between the organism and its environment. As such, the skin acts as a physical and an immunological barrier to protect the body from dangerous substances and pathogens (74). However, the skin can also be easily wounded and then becomes a main entry route for foreign pathogens. Again, it is highly conceivable that the mechanisms of tissue regeneration, including HGF/Met signaling, are interrelated with immune regulatory mechanisms. Surprisingly, there are only a few studies revealing a role of the HGF/Met signaling pathway in skin injury and inflammation.

A study employing a mouse model with conditionally disrupted Met gene in epidermal keratinocytes revealed an indispensable role for the HGF–Met pathway in skin wound healing (75). Particularly, Met-deficient epidermal keratinocytes failed to restore skin wound re-epithelialization, while other growth factors and bioactive molecules were functional. Further studies provided additional mechanistic insights and thus corroborated the role of Met signaling in keratinocytes for wound healing (76–78). Interestingly, keratinocytes are a source of HGF upon skin injury in humans (79).

A previous study identified dermal fibroblasts as a major source of HGF in skin upon infection or stimulation with pro-inflammatory cytokines (80) indicating a role for HGF/Met signaling also in dermal tissue homeostasis. Indeed, in the tight-skin mouse, a genetic model of human systemic sclerosis HGF was shown to ameliorate dermal sclerosis (81). The tight-skin mouse model exhibits fibrosis and thickening of subcutaneous dermal tissue, which was diminished upon HGF treatment. Exogenous HGF was found to suppress expression of IL-4 and TGF-β mRNA (81), which has been suggested to impact on fibrogenesis and in the hypodermal thickness of tight-skin mice (81, 82). In particular, HGF was found to inhibit the production of IL-4 in CD4+ T cells stimulated by allogeneic DCs, and it is tempting to speculate that this was due to Met-mediated activity of HGF on DCs.

Clear evidence for the role of Met signaling in skin DCs came again from studies using a conditional Met-knockout mouse model (83) in which DC-dependent contact hypersensitivity (CHS) reactions were addressed (53). Skin DC populations including LCs were found to express Met, and HGF stimulation effectively activated Met signaling and induced LC emigration from skin (Figure 2) (52, 53). By contrast, skin-resident DCs in Met-deficient mice upon activation failed to emigrate from skin toward the draining LNs although DCs displayed an activated phenotype (53). Consequently, Met-deficiency resulted in strongly impaired CHS reactions in response to contact allergens, which could be also achieved by pharmacological inhibition of Met signaling in wild-type control mice. Emigration of resident LCs from the skin upon stimulation requires a multitude of tissue remodeling capacities that allows detachment from surrounding tissue, adherence to and migration through ECM, and crossing tissue boundaries. Met signaling was found essential in migration of BM-derived DCs through ECM that requires matrix metalloproteinase (MMP) activities for matrix degradation. Indeed, proteolytic activity of both MMP-2 and MMP-9 was found regulated by Met in BM-derived DCs (53), in line with previous studies that revealed a critical role of MMP-2 and MMP-9 in LC migration (Figure 2) (84–86). In summary, these findings established Met signaling as a key mechanism of LC detachment from the epidermal tissue and emigration from the skin upon activation.

## MET-Driven EMT in LCs as a Regulator of Skin Immunity

The regulation of LC mobilization and migration by HGF/Met signaling upon inflammatory activation results in a series of phenotypic conversions comprising, e.g., detachment from surrounding tissue and activation of MMPs resulting in interstitial migration and crossing of tissue boundaries. Collectively, all these phenotypic alterations have a striking analogy to a Met signal-driven mechanism identified during embryonic development, wound healing, and invasive growth of tumors known as EMT (8, 13, 14, 87–89). The genetic program underlying this process leads to the transient conversion of immobile epithelial cells into a migratory mesenchymal phenotype. Thus, we propose the concept that a genetic program similar to EMT is accomplished by Met signaling in LCs (29, 53). Similar to Met-driven EMT of epithelial cells, LCs need to disrupt their physical contact to neighboring cells mediated by adherens and tight junctions. A major molecular hallmark of EMT is the loss of E-cadherin expression. EMT is further characterized by the downregulation of various other factors involved in formation of adherens and tight junction structures, including zonula occludens (ZO) proteins, cytokeratins, occludins, claudins, and EpCAM leading to the disassembly of cell-to-cell contacts (88–90). Cells simultaneously acquire a mesenchymal phenotype, including the expression of N-cadherin, vimentin, integrins, and MMPs and reorganization of their cytoskeleton, which collectively enable cell migration. Again, it has been well recognized that the Met-driven stimulation of proteolytic MMP activity advances tumor cell dissociation and scattering (87–89). The EMT program is controlled by an intricate network of transcriptional regulators including basic helix–loop–helix factors (e.g., Twist1) and zinc finger and E-box binding proteins (ZEB) 1 and 2 [reviewed in Ref. (89–91)].

Langerhans cells in skin express a broad range of epithelial-like adhesive molecules that permit the functional integration into the keratinocyte layer. This includes tight junction proteins, such as claudin-1 and ZO-1 (92, 93), which have been shown to maintain tight junction integrity during antigen uptake (93). Furthermore, human LCs derived in a well-established *in vitro* model showed in addition expression of occludin, ZO-3, JAM1, and cytokeratins (CK8 and CK18) (94), and it is highly conceivable that this is also true *in vivo*. LCs also express adherens junction proteins that mediate homophilic binding to other cells, including E-cadherin, EpCAM/TROP1, and TROP2 (95–97), and the specific impacts of E-cadherin and EpCAM on LC motility, migration, and function have been well recognized (98–100). Remarkably, the maturation of activated LCs toward a migratory phenotype revealed downregulation of E-cadherin and EpCAM, accompanied by upregulated expression of N-cadherin and the EMT regulators ZEB1 and ZEB2 (98, 101, 102). These findings, together with the regulation of MMPs in DCs described earlier, support the notion that a Met-driven EMT program is accomplished after LCs are activated (29).

## Concluding Remarks

In summary, Met signaling in skin resident DCs including LCs appears to be a critical determinant for maintaining normal immune function and as an important constituent that interlaces tissue regenerative functions with the appropriate immune responses that must be accomplished after tissue injury, infection, or inflammation. Other studies suggest a protective role of HGF/Met signaling against autoimmunity by directing DCs toward a tolerogenic phenotype. This and a number of further activities of HGF/Met signaling on other immune cells suggest the HGF/Met pathway as a potential target for treatment of inflammatory and autoimmune disorders, including skin diseases and transplantation (103, 104). Due to the critical role of Met signaling for tumor invasion and metastasis, drug targeting of the Met receptor and/or pathways is highly considered as a potential means for therapy of a number of epithelial cancers. Consequently, attempts to block Met-induced migration of tumor cells may lead to altered immune functions in cancer patients and thus possibly to increased susceptibility to infection and/or development of autoimmune disorders. Conversely, approaches to promote immune tolerance *via* HGF/Met in immune cells could concurrently stimulate potential tumor cells toward invasive growth. The knowledge of the HGF/Met signaling mechanisms in DCs is still in its infancy and must be extended to (i) develop save Met-based therapies in the future and (ii) corroborate the concept that a Met-driven execution of an EMT program in DCs is indeed a generic mechanism.

## Author Contributions

All authors listed have made a substantial, direct, and intellectual contribution to the work and approved it for publication.

## Conflict of Interest Statement

The authors declare that the research was conducted in the absence of any commercial or financial relationships that could be construed as a potential conflict of interest.

## References

[1] Nakamura T Nawa K Ichihara A. Partial purification and characterization of hepatocyte growth factor from serum of hepatectomized rats. Biochem Biophys Res Commun (1984) 122(3):1450–9. 10.1016/0006-291X(84)91253-1 6477569

[2] Russell WE McGowan JA Bucher NL. Partial characterization of a hepatocyte growth factor from rat platelets. J Cell Physiol (1984) 119(2):183–92. 10.1002/jcp.1041190207 6715416

[3] Stoker M Gherardi E Perryman M Gray J. Scatter factor is a fibroblast-derived modulator of epithelial cell mobility. Nature (1987) 327(6119):239–42. 10.1038/327239a0 2952888

[4] Shima N Nagao M Ogaki F Tsuda E Murakami A Higashio K. Tumor cytotoxic factor/hepatocyte growth factor from human fibroblasts: cloning of its cDNA, purification and characterization of recombinant protein. Biochem Biophys Res Commun (1991) 180(2):1151–8. 10.1016/S0006-291X(05)81187-8 1835383

[5] Nakamura T Sakai K Nakamura T Matsumoto K. Hepatocyte growth factor twenty years on: much more than a growth factor. J Gastroenterol Hepatol (2011) 26(Suppl 1):188–202. 10.1111/j.1440-1746.2010.06549.x 21199531

[6] Cooper CS Park M Blair DG Tainsky MA Huebner K Croce CM Molecular cloning of a new transforming gene from a chemically transformed human cell line. Nature (1984) 311(5981):29–33. 10.1038/311029a0 6590967

[7] Bottaro DP Rubin JS Faletto DL Chan AM Kmiecik TE Vande Woude GF Identification of the hepatocyte growth factor receptor as the c-met proto-oncogene product. Science (1991) 251(4995):802–4. 10.1126/science.1846706 1846706

[8] Birchmeier C Birchmeier W Gherardi E Vande Woude GF Met, metastasis, motility and more. Nat Rev Mol Cell Biol (2003) 4(12):915–25. 10.1038/nrm1261 14685170

[9] Schmidt C Bladt F Goedecke S Brinkmann V Zschiesche W Sharpe M Scatter factor/hepatocyte growth factor is essential for liver development. Nature (1995) 373(6516):699–702. 10.1038/373699a0 7854452

[10] Bladt F Riethmacher D Isenmann S Aguzzi A Birchmeier C. Essential role for the c-met receptor in the migration of myogenic precursor cells into the limb bud. Nature (1995) 376(6543):768–71. 10.1038/376768a0 7651534

[11] Uehara Y Minowa O Mori C Shiota K Kuno J Noda T Placental defect and embryonic lethality in mice lacking hepatocyte growth factor/scatter factor. Nature (1995) 373(6516):702–5. 10.1038/373702a0 7854453

[12] Dietrich S Abou-Rebyeh F Brohmann H Bladt F Sonnenberg-Riethmacher E Yamaai T The role of SF/HGF and c-Met in the development of skeletal muscle. Development (1999) 126(8):1621–9. 10079225 10.1242/dev.126.8.1621

[13] Boccaccio C Comoglio PM. Invasive growth: a MET-driven genetic programme for cancer and stem cells. Nat Rev Cancer (2006) 6(8):637–45. 10.1038/nrc1912 16862193

[14] Trusolino L Bertotti A Comoglio PM. MET signalling: principles and functions in development, organ regeneration and cancer. Nat Rev Mol Cell Biol (2010) 11(12):834–48. 10.1038/nrm3012 21102609

[15] Knudsen BS Vande Woude G. Showering c-MET-dependent cancers with drugs. Curr Opin Genet Dev (2008) 18(1):87–96. 10.1016/j.gde.2008.02.001 18406132

[16] Comoglio PM Giordano S Trusolino L. Drug development of MET inhibitors: targeting oncogene addiction and expedience. Nat Rev Drug Discov (2008) 7(6):504–16. 10.1038/nrd2530 18511928

[17] Gherardi E Birchmeier W Birchmeier C Vande Woude G. Targeting MET in cancer: rationale and progress. Nat Rev Cancer (2012) 12(2):89–103. 10.1038/nrc3205 22270953

[18] Sakai K Aoki S Matsumoto K. Hepatocyte growth factor and Met in drug discovery. J Biochem (2015) 157(5):271–84. 10.1093/jb/mvv027 25770121

[19] Donate LE Gherardi E Srinivasan N Sowdhamini R Aparicio S Blundell TL Molecular evolution and domain structure of plasminogen-related growth factors (HGF/SF and HGF1/MSP). Protein Sci (1994) 3(12):2378–94. 10.1002/pro.5560031222 7756992 PMC2142779

[20] Lokker NA Mark MR Luis EA Bennett GL Robbins KA Baker JB Structure-function analysis of hepatocyte growth factor: identification of variants that lack mitogenic activity yet retain high affinity receptor binding. EMBO J (1992) 11(7):2503–10. 1321034 10.1002/j.1460-2075.1992.tb05315.x PMC556725

[21] Matsumoto K Nakamura T. Emerging multipotent aspects of hepatocyte growth factor. J Biochem (1996) 119(4):591–600. 10.1093/oxfordjournals.jbchem.a021283 8743556

[22] Matsumoto K Kataoka H Date K Nakamura T. Cooperative interaction between alpha- and beta-chains of hepatocyte growth factor on c-Met receptor confers ligand-induced receptor tyrosine phosphorylation and multiple biological responses. J Biol Chem (1998) 273(36):22913–20. 10.1074/jbc.273.36.22913 9722511

[23] Kobayashi T Honke K Miyazaki T Matsumoto K Nakamura T Ishizuka I Hepatocyte growth factor specifically binds to sulfoglycolipids. J Biol Chem (1994) 269(13):9817–21. 8144574

[24] Shimomura T Kondo J Ochiai M Naka D Miyazawa K Morimoto Y Activation of the zymogen of hepatocyte growth factor activator by thrombin. J Biol Chem (1993) 268(30):22927–32. 8226803

[25] Miyazawa K Shimomura T Kitamura N. Activation of hepatocyte growth factor in the injured tissues is mediated by hepatocyte growth factor activator. J Biol Chem (1996) 271(7):3615–8. 10.1074/jbc.271.7.3615 8631970

[26] Kataoka H Kawaguchi M. Hepatocyte growth factor activator (HGFA): pathophysiological functions in vivo. FEBS J (2010) 277(10):2230–7. 10.1111/j.1742-4658.2010.07640.x 20402763

[27] Hashimoto S Suzuki T Dong HY Nagai S Yamazaki N Matsushima K. Serial analysis of gene expression in human monocyte-derived dendritic cells. Blood (1999) 94(3):845–52. 10419874

[28] Manning G Whyte DB Martinez R Hunter T Sudarsanam S. The protein kinase complement of the human genome. Science (2002) 298(5600):1912–34. 10.1126/science.1075762 12471243

[29] Hieronymus T Zenke M Baek JH Sere K. The clash of Langerhans cell homeostasis in skin: should I stay or should I go? Semin Cell Dev Biol (2015) 41:30–8. 10.1016/j.semcdb.2014.02.009 24613914

[30] Gherardi E Youles ME Miguel RN Blundell TL Iamele L Gough J Functional map and domain structure of MET, the product of the c-met protooncogene and receptor for hepatocyte growth factor/scatter factor. Proc Natl Acad Sci U S A (2003) 100(21):12039–44. 10.1073/pnas.2034936100 14528000 PMC218709

[31] Rosario M Birchmeier W. How to make tubes: signaling by the Met receptor tyrosine kinase. Trends Cell Biol (2003) 13(6):328–35. 10.1016/S0962-8924(03)00104-1 12791299

[32] Trusolino L Bertotti A Comoglio PM. A signaling adapter function for alpha6beta4 integrin in the control of HGF-dependent invasive growth. Cell (2001) 107(5):643–54. 10.1016/S0092-8674(01)00567-0 11733063

[33] Giordano S Corso S Conrotto P Artigiani S Gilestro G Barberis D The semaphorin 4D receptor controls invasive growth by coupling with Met. Nat Cell Biol (2002) 4(9):720–4. 10.1038/ncb843 12198496

[34] van der Voort R Taher TE Wielenga VJ Spaargaren M Prevo R Smit L Heparan sulfate-modified CD44 promotes hepatocyte growth factor/scatter factor-induced signal transduction through the receptor tyrosine kinase c-Met. J Biol Chem (1999) 274(10):6499–506. 10.1074/jbc.274.10.6499 10037743

[35] Orian-Rousseau V Chen L Sleeman JP Herrlich P Ponta H. CD44 is required for two consecutive steps in HGF/c-Met signaling. Genes Dev (2002) 16(23):3074–86. 10.1101/gad.242602 12464636 PMC187488

[36] Gordin M Tesio M Cohen S Gore Y Lantner F Leng L c-Met and its ligand hepatocyte growth factor/scatter factor regulate mature B cell survival in a pathway induced by CD74. J Immunol (2010) 185(4):2020–31. 10.4049/jimmunol.0902566 20639480 PMC3646513

[37] Matteucci E Ridolfi E Desiderio MA. Hepatocyte growth factor differently influences Met-E-cadherin phosphorylation and downstream signaling pathway in two models of breast cells. Cell Mol Life Sci (2006) 63(17):2016–26. 10.1007/s00018-006-6137-0 16909210 PMC11136117

[38] Conrotto P Corso S Gamberini S Comoglio PM Giordano S. Interplay between scatter factor receptors and B plexins controls invasive growth. Oncogene (2004) 23(30):5131–7. 10.1038/sj.onc.1207650 15184888

[39] Walzer T Galibert L De Smedt T. Dendritic cell function in mice lacking plexin C1. Int Immunol (2005) 17(7):943–50. 10.1093/intimm/dxh274 15967782

[40] Takamatsu H Takegahara N Nakagawa Y Tomura M Taniguchi M Friedel RH Semaphorins guide the entry of dendritic cells into the lymphatics by activating myosin II. Nat Immunol (2010) 11(7):594–600. 10.1038/ni.1885 20512151 PMC3045806

[41] Roney KE O’Connor BP Wen H Holl EK Guthrie EH Davis BK Plexin-B2 negatively regulates macrophage motility, Rac, and Cdc42 activation. PLoS One (2011) 6(9):e24795. 10.1371/journal.pone.0024795 21966369 PMC3179467

[42] Kmiecik TE Keller JR Rosen E Vande Woude GF. Hepatocyte growth factor is a synergistic factor for the growth of hematopoietic progenitor cells. Blood (1992) 80(10):2454–7. 1421367

[43] Zarnegar R Michalopoulos GK The many faces of hepatocyte growth factor: from hepatopoiesis to hematopoiesis. J Cell Biol (1995) 129(5):1177–80. 10.1083/jcb.129.5.1177 7775566 PMC2120475

[44] Hubel J Hieronymus T. HGF/Met-signaling contributes to immune regulation by modulating tolerogenic and motogenic properties of dendritic cells. Biomedicines (2015) 3(1):138–48. 10.3390/biomedicines3010138 28536404 PMC5344228

[45] Finisguerra V Di Conza G Di Matteo M Serneels J Costa S Thompson AA MET is required for the recruitment of anti-tumoural neutrophils. Nature (2015) 522(7556):349–53. 10.1038/nature14407 25985180 PMC4594765

[46] Benkhoucha M Molnarfi N Kaya G Belnoue E Bjarnadottir K Dietrich PY Identification of a novel population of highly cytotoxic c-Met-expressing CD8+ T lymphocytes. EMBO Rep (2017) 18(9):1545–58. 10.15252/embr.201744075 28751311 PMC5579394

[47] van der Voort R Taher TE Keehnen RM Smit L Groenink M Pals ST. Paracrine regulation of germinal center B cell adhesion through the c-met-hepatocyte growth factor/scatter factor pathway. J Exp Med (1997) 185(12):2121–31. 10.1084/jem.185.12.2121 9182684 PMC2196350

[48] Beilmann M Vande Woude GF Dienes HP Schirmacher P. Hepatocyte growth factor-stimulated invasiveness of monocytes. Blood (2000) 95(12):3964–9. 10845935

[49] Galimi F Cottone E Vigna E Arena N Boccaccio C Giordano S Hepatocyte growth factor is a regulator of monocyte-macrophage function. J Immunol (2001) 166(2):1241–7. 10.4049/jimmunol.166.2.1241 11145707

[50] Okunishi K Dohi M Nakagome K Tanaka R Mizuno S Matsumoto K A novel role of hepatocyte growth factor as an immune regulator through suppressing dendritic cell function. J Immunol (2005) 175(7):4745–53. 10.4049/jimmunol.175.7.4745 16177122

[51] Benkhoucha M Santiago-Raber ML Schneiter G Chofflon M Funakoshi H Nakamura T Hepatocyte growth factor inhibits CNS autoimmunity by inducing tolerogenic dendritic cells and CD25+Foxp3+ regulatory T cells. Proc Natl Acad Sci U S A (2010) 107(14):6424–9. 10.1073/pnas.0912437107 20332205 PMC2851995

[52] Kurz SM Diebold SS Hieronymus T Gust TC Bartunek P Sachs M The impact of c-met/scatter factor receptor on dendritic cell migration. Eur J Immunol (2002) 32(7):1832–8. 10.1002/1521-4141(200207)32:7<1832::AID-IMMU1832>3.0.CO;2-2 12115601

[53] Baek JH Birchmeier C Zenke M Hieronymus T. The HGF receptor/Met tyrosine kinase is a key regulator of dendritic cell migration in skin immunity. J Immunol (2012) 189(4):1699–707. 10.4049/jimmunol.1200729 22802413

[54] Skibinski G Skibinska A James K. The role of hepatocyte growth factor and its receptor c-met in interactions between lymphocytes and stromal cells in secondary human lymphoid organs. Immunology (2001) 102(4):506–14. 10.1046/j.1365-2567.2001.01186.x 11328385 PMC1783204

[55] Tamura S Sugawara T Tokoro Y Taniguchi H Fukao K Nakauchi H Expression and function of c-Met, a receptor for hepatocyte growth factor, during T-cell development. Scand J Immunol (1998) 47(4):296–301. 10.1046/j.1365-3083.1998.00324.x 9600310

[56] Takai K Hara J Matsumoto K Hosoi G Osugi Y Tawa A Hepatocyte growth factor is constitutively produced by human bone marrow stromal cells and indirectly promotes hematopoiesis. Blood (1997) 89(5):1560–5. 9057637

[57] Kuroiwa T Iwasaki T Imado T Sekiguchi M Fujimoto J Sano H. Hepatocyte growth factor prevents lupus nephritis in a murine lupus model of chronic graft-versus-host disease. Arthritis Res Ther (2006) 8(4):R123. 10.1186/ar2012 16859527 PMC1779408

[58] Okunishi K Dohi M Fujio K Nakagome K Tabata Y Okasora T Hepatocyte growth factor significantly suppresses collagen-induced arthritis in mice. J Immunol (2007) 179(8):5504–13. 10.4049/jimmunol.179.8.5504 17911637

[59] Singhal E Kumar P Sen P. A novel role for Bruton’s tyrosine kinase in hepatocyte growth factor-mediated immunoregulation of dendritic cells. J Biol Chem (2011) 286(37):32054–63. 10.1074/jbc.M111.271247 21784852 PMC3173196

[60] Tamura M Arakaki N Tsubouchi H Takada H Daikuhara Y. Enhancement of human hepatocyte growth factor production by interleukin-1 alpha and -1 beta and tumor necrosis factor-alpha by fibroblasts in culture. J Biol Chem (1993) 268(11):8140–5. 7681834

[61] Liu Y Michalopoulos GK Zarnegar R. Structural and functional characterization of the mouse hepatocyte growth factor gene promoter. J Biol Chem (1994) 269(6):4152–60. 8307976

[62] Gohda E Kataoka H Tsubouchi H Daikilara Y Yamamoto I. Phorbol ester-induced secretion of human hepatocyte growth factor by human skin fibroblasts and its inhibition by dexamethasone. FEBS Lett (1992) 301(1):107–10. 10.1016/0014-5793(92)80220-B 1451778

[63] Inaba M Koyama H Hino M Okuno S Terada M Nishizawa Y Regulation of release of hepatocyte growth factor from human promyelocytic leukemia cells, HL-60, by 1,25-dihydroxyvitamin D3, 12-O-tetradecanoylphorbol 13-acetate, and dibutyryl cyclic adenosine monophosphate. Blood (1993) 82(1):53–9. 8391878

[64] Gohda E Matsunaga T Kataoka H Yamamoto I. TGF-beta is a potent inhibitor of hepatocyte growth factor secretion by human fibroblasts. Cell Biol Int Rep (1992) 16(9):917–26. 10.1016/S0309-1651(06)80171-2 1423659

[65] Jiang Q Azuma E Tanaka M Kobayashi M Hirayama M Kumamoto T Differential responsiveness of cord and adult blood monocytes to hepatocyte growth factor. Clin Exp Immunol (2001) 125(2):222–8. 10.1046/j.1365-2249.2001.01591.x 11529913 PMC1906136

[66] Moransard M Sawitzky M Fontana A Suter T. Expression of the HGF receptor c-met by macrophages in experimental autoimmune encephalomyelitis. Glia (2010) 58(5):559–71. 10.1002/glia.20945 19941340

[67] Oh K Iimuro Y Takeuchi M Kaneda Y Iwasaki T Terada N Ameliorating effect of hepatocyte growth factor on inflammatory bowel disease in a murine model. Am J Physiol Gastrointest Liver Physiol (2005) 288(4):G729–35. 10.1152/ajpgi.00438.2004 15550554

[68] Hanawa T Suzuki K Kawauchi Y Takamura M Yoneyama H Han GD Attenuation of mouse acute colitis by naked hepatocyte growth factor gene transfer into the liver. J Gene Med (2006) 8(5):623–35. 10.1002/jgm.880 16479533

[69] Benkhoucha M Molnarfi N Dunand-Sauthier I Merkler D Schneiter G Bruscoli S Hepatocyte growth factor limits autoimmune neuroinflammation via glucocorticoid-induced leucine zipper expression in dendritic cells. J Immunol (2014) 193(6):2743–52. 10.4049/jimmunol.1302338 25114100

[70] Singhal E Sen P Hepatocyte growth factor-induced c-Src-phosphatidylinositol 3-kinase-AKT-mammalian target of rapamycin pathway inhibits dendritic cell activation by blocking IkappaB kinase activity. Int J Biochem Cell Biol (2011) 43(8):1134–46. 10.1016/j.biocel.2011.04.006 21536148

[71] Corinti S Albanesi C la Sala A Pastore S Girolomoni G. Regulatory activity of autocrine IL-10 on dendritic cell functions. J Immunol (2001) 166(7):4312–8. 10.4049/jimmunol.166.7.4312 11254683

[72] Cohen N Mouly E Hamdi H Maillot MC Pallardy M Godot V GILZ expression in human dendritic cells redirects their maturation and prevents antigen-specific T lymphocyte response. Blood (2006) 107(5):2037–44. 10.1182/blood-2005-07-2760 16293609

[73] Hamdi H Godot V Maillot MC Prejean MV Cohen N Krzysiek R Induction of antigen-specific regulatory T lymphocytes by human dendritic cells expressing the glucocorticoid-induced leucine zipper. Blood (2007) 110(1):211–9. 10.1182/blood-2006-10-052506 17356131 PMC2077304

[74] Pasparakis M Haase I Nestle FO. Mechanisms regulating skin immunity and inflammation. Nat Rev Immunol (2014) 14(5):289–301. 10.1038/nri3646 24722477

[75] Chmielowiec J Borowiak M Morkel M Stradal T Munz B Werner S c-Met is essential for wound healing in the skin. J Cell Biol (2007) 177(1):151–62. 10.1083/jcb.200701086 17403932 PMC2064119

[76] Xu Y Xia W Baker D Zhou J Cha HC Voorhees JJ Receptor-type protein tyrosine phosphatase beta (RPTP-beta) directly dephosphorylates and regulates hepatocyte growth factor receptor (HGFR/Met) function. J Biol Chem (2011) 286(18):15980–8. 10.1074/jbc.M110.212597 21454675 PMC3091207

[77] Singh A Nascimento JM Kowar S Busch H Boerries M. Boolean approach to signalling pathway modelling in HGF-induced keratinocyte migration. Bioinformatics (2012) 28(18):i495–501. 10.1093/bioinformatics/bts410 22962472 PMC3436837

[78] Miura Y Ngo Thai Bich V Furuya M Hasegawa H Takahashi S Katagiri N The small G protein Arf6 expressed in keratinocytes by HGF stimulation is a regulator for skin wound healing. Sci Rep (2017) 7:46649. 10.1038/srep46649 28429746 PMC5399375

[79] Nayeri F Xu J Abdiu A Nayeri T Aili D Liedberg B Autocrine production of biologically active hepatocyte growth factor (HGF) by injured human skin. J Dermatol Sci (2006) 43(1):49–56. 10.1016/j.jdermsci.2006.03.004 16621453

[80] Matsumoto K Okazaki H Nakamura T. Up-regulation of hepatocyte growth factor gene expression by interleukin-1 in human skin fibroblasts. Biochem Biophys Res Commun (1992) 188(1):235–43. 10.1016/0006-291X(92)92375-8 1384479

[81] Iwasaki T Imado T Kitano S Sano H. Hepatocyte growth factor ameliorates dermal sclerosis in the tight-skin mouse model of scleroderma. Arthritis Res Ther (2006) 8(6):R161. 10.1186/ar2068 17049072 PMC1794503

[82] Blank M Levy Y Amital H Shoenfeld Y Pines M Genina O The role of intravenous immunoglobulin therapy in mediating skin fibrosis in tight skin mice. Arthritis Rheum (2002) 46(6):1689–90. 10.1002/art.10363 12115202

[83] Borowiak M Garratt AN Wustefeld T Strehle M Trautwein C Birchmeier C. Met provides essential signals for liver regeneration. Proc Natl Acad Sci U S A (2004) 101(29):10608–13. 10.1073/pnas.0403412101 15249655 PMC490025

[84] Ratzinger G Stoitzner P Ebner S Lutz MB Layton GT Rainer C Matrix metalloproteinases 9 and 2 are necessary for the migration of Langerhans cells and dermal dendritic cells from human and murine skin. J Immunol (2002) 168(9):4361–71. 10.4049/jimmunol.168.9.4361 11970978

[85] Yen JH Khayrullina T Ganea D. PGE2-induced metalloproteinase-9 is essential for dendritic cell migration. Blood (2008) 111(1):260–70. 10.1182/blood-2007-05-090613 17925490 PMC2200811

[86] Saalbach A Klein C Schirmer C Briest W Anderegg U Simon JC. Dermal fibroblasts promote the migration of dendritic cells. J Invest Dermatol (2010) 130(2):444–54. 10.1038/jid.2009.253 19710690

[87] Thiery JP Acloque H Huang RY Nieto MA Epithelial-mesenchymal transitions in development and disease. Cell (2009) 139(5):871–90. 10.1016/j.cell.2009.11.007 19945376

[88] Trusolino L Comoglio PM. Scatter-factor and semaphorin receptors: cell signalling for invasive growth. Nat Rev Cancer (2002) 2(4):289–300. 10.1038/nrc779 12001990

[89] Christofori G. New signals from the invasive front. Nature (2006) 441(7092):444–50. 10.1038/nature04872 16724056

[90] Peinado H Olmeda D Cano A. Snail, Zeb and bHLH factors in tumour progression: an alliance against the epithelial phenotype? Nat Rev Cancer (2007) 7(6):415–28. 10.1038/nrc2131 17508028

[91] Lamouille S Subramanyam D Blelloch R Derynck R. Regulation of epithelial-mesenchymal and mesenchymal-epithelial transitions by microRNAs. Curr Opin Cell Biol (2013) 25(2):200–7. 10.1016/j.ceb.2013.01.008 23434068 PMC4240277

[92] Zimmerli SC Hauser C. Langerhans cells and lymph node dendritic cells express the tight junction component claudin-1. J Invest Dermatol (2007) 127(10):2381–90. 10.1038/sj.jid.5700882 17508021

[93] Kubo A Nagao K Yokouchi M Sasaki H Amagai M. External antigen uptake by Langerhans cells with reorganization of epidermal tight junction barriers. J Exp Med (2009) 206(13):2937–46. 10.1084/jem.20091527 19995951 PMC2806471

[94] Yasmin N Konradi S Eisenwort G Schichl YM Seyerl M Bauer T Beta-catenin promotes the differentiation of epidermal Langerhans dendritic cells. J Invest Dermatol (2013) 133(5):1250–9. 10.1038/jid.2012.481 23303458

[95] Tang A Amagai M Granger LG Stanley JR Udey MC. Adhesion of epidermal Langerhans cells to keratinocytes mediated by E-cadherin. Nature (1993) 361(6407):82–5. 10.1038/361082a0 8421498

[96] Borkowski TA Nelson AJ Farr AG Udey MC. Expression of gp40, the murine homologue of human epithelial cell adhesion molecule (Ep-CAM), by murine dendritic cells. Eur J Immunol (1996) 26(1):110–4. 10.1002/eji.1830260117 8566052

[97] Eisenwort G Jurkin J Yasmin N Bauer T Gesslbauer B Strobl H Identification of TROP2 (TACSTD2), an EpCAM-like molecule, as a specific marker for TGF-beta1-dependent human epidermal Langerhans cells. J Invest Dermatol (2011) 131(10):2049–57. 10.1038/jid.2011.164 21677668

[98] Schwarzenberger K Udey MC. Contact allergens and epidermal proinflammatory cytokines modulate Langerhans cell E-cadherin expression in situ. J Invest Dermatol (1996) 106(3):553–8. 10.1111/1523-1747.ep12344019 8648193

[99] Gaiser MR Lammermann T Feng X Igyarto BZ Kaplan DH Tessarollo L Cancer-associated epithelial cell adhesion molecule (EpCAM; CD326) enables epidermal Langerhans cell motility and migration in vivo. Proc Natl Acad Sci U S A (2012) 109(15):E889–97. 10.1073/pnas.1117674109 22411813 PMC3326512

[100] Kashem SW Haniffa M Kaplan DH. Antigen-presenting cells in the skin. Annu Rev Immunol (2017) 35:469–99. 10.1146/annurev-immunol-051116-052215 28226228

[101] Bobr A Igyarto BZ Haley KM Li MO Flavell RA Kaplan DH Autocrine/paracrine TGF-beta1 inhibits Langerhans cell migration. Proc Natl Acad Sci U S A (2012) 109(26):10492–7. 10.1073/pnas.1119178109 22689996 PMC3387113

[102] Konradi S Yasmin N Haslwanter D Weber M Gesslbauer B Sixt M Langerhans cell maturation is accompanied by induction of N-cadherin and the transcriptional regulators of epithelial-mesenchymal transition ZEB1/2. Eur J Immunol (2013) 44(2):553–60. 10.1002/eji.201343681 24165969

[103] Molnarfi N Benkhoucha M Funakoshi H Nakamura T Lalive PH. Hepatocyte growth factor: a regulator of inflammation and autoimmunity. Autoimmun Rev (2015) 14(4):293–303. 10.1016/j.autrev.2014.11.013 25476732

[104] Ilangumaran S Villalobos-Hernandez A Bobbala D Ramanathan S. The hepatocyte growth factor (HGF)-MET receptor tyrosine kinase signaling pathway: diverse roles in modulating immune cell functions. Cytokine (2016) 82:125–39. 10.1016/j.cyto.2015.12.013 26822708

